# Metal interference screw fixation combinations show high revision rates in primary hamstring tendon ACL reconstruction

**DOI:** 10.1186/s12891-023-07109-y

**Published:** 2024-01-02

**Authors:** Janina Kaarre, Neilen A. Benvegnu, Ian D. Engler, Ehab M. Nazzal, Bálint Zsidai, Eric Hamrin Senorski, Volker Musahl, Kristian Samuelsson

**Affiliations:** 1 Department of Orthopaedics, Institute of Clinical Sciences, Sahlgrenska Academy, University of Gothenburg, Gothenburg, Sweden; 2 Sahlgrenska Sports Medicine Center, Gothenburg, Sweden; 3 Department of Orthopaedic Surgery, UPMC Freddie Fu Sports Medicine Center, University of Pittsburgh, Pittsburgh, USA; 4 Central Maine Healthcare Orthopedics, Central Maine Medical Center, Auburn, ME USA; 5 Department of Health and Rehabilitation, Institute of Neuroscience and Physiology, Sahlgrenska Academy, University of Gothenburg, Gothenburg, Sweden; 6 Department of Orthopaedics, Sahlgrenska University Hospital, Mölndal, Sweden

**Keywords:** ACL, Anterior cruciate ligament, Reconstruction, Hamstring tendon autograft, Fixation, Revision

## Abstract

**Background:**

Different fixation methods in anterior cruciate ligament reconstruction (ACLR) have been associated with different revision rates, specifically in the early postoperative period. However, most previous research has either grouped together different fixation types or evaluated femoral-sided fixation or tibial-sided fixation separately. Therefore, the purpose of this study was to determine ACL revision rates for specific combinations of femoral and tibial fixation methods within 2 years of primary hamstring tendon autograft ACLR based on data from the Swedish National Knee Ligament Registry (SNKLR).

**Methods:**

Patients that underwent primary hamstring tendon autograft ACLR between 2005 and 2018 in the SNKLR were included. The collected data included patient characteristics (age, sex, body mass index [BMI]), activity at time of injury, surgical information (concomitant injuries, time from injury to surgery, fixation types at the femur and tibia), and subsequent revision ACLR. Revision rate within 2 years of the index procedure was chosen, as ACLR fixation is most likely to contribute to ACLR revision within the first 2 years, during graft maturation.

**Results:**

Of the 23,238 included patients undergoing primary hamstring ACLR, 581 (2.5%) underwent revision ACLR within 2 years of the index procedure. Among the combinations used for > 300 patients, the femoral metal interference screw/tibial metal interference screw fixation combination had the highest revision rate followed by metal interference screw/resorbable screw and Endobutton/AO screw fixation combinations, with respective revision rates of 4.0, 3.0, and 3.0%. The lowest revision rate within 2 years of ACLR was found in the Endobutton/metal interference screw with backup Osteosuture fixation combination, used in 433 cases, with a failure rate of 0.9%.

**Conclusion:**

Different early ACL revision rates were found across different combinations of femoral and tibial fixation devices within 2 years of primary hamstring tendon autograft ACLR. Metal interference screw fixation, particularly when performed on both the femoral and tibial sides, most frequently resulted in revision ACLR. These findings may be helpful for surgeons in selecting appropriate fixation devices for hamstring ACLR.

**Level of evidence:**

IV

**Supplementary Information:**

The online version contains supplementary material available at 10.1186/s12891-023-07109-y.

## Introduction

Anterior cruciate ligament reconstruction (ACLR) revision is one of the most thoroughly researched topics in sports medicine, and yet there remain unanswered questions. Several factors contributing to ACL revision risk have been identified, including patient-, technical-, and injury-related factors [1–4]. Two often discussed technical decisions impacting ACLR revision rates are graft choice and fixation method [3, 5]. While by far the most frequently used graft type in Sweden continues to be hamstring tendon autograft, preferences regarding fixation vary widely among surgeons and healthcare institutions [6]. As a result of consistency in graft choice among Swedish surgeons, short-term outcomes may instead depend upon the method of graft fixation [7–9].

Fixation methods in ACLR have been shown to impact revision rate, specifically in the early postoperative period [7, 10]. Most prior literature has either grouped together femoral and tibia-sided fixation types or evaluated femoral-sided fixation or tibial-sided fixation separately [5, 11, 12]. However, previous studies have not investigated the role of specific femoral and tibial fixation combinations on ACLR revision rates within the early postoperative period. Analyzing the interaction between fixation devices on both the femoral and tibial sides is important, as their effect may be complementary or contradictory, which cannot be fully comprehended by evaluating each tibial and femoral fixation type separately.

The purpose of this study was to determine the rate of ACL revision with respect to specific combinations of femoral and tibial fixation devices within 2 years following primary hamstring tendon autograft ACLR based on data from the Swedish National Knee Ligament Registry (SNKLR).

## Methods

This registry study was performed in accordance with the Declaration of Helsinki and was approved by the Regional Ethical Board in Stockholm, Sweden (2011/337–31/3), and the Swedish Ethical Review Authority (2022–00913-01).

The data source was the SNKLR, a national registry of cruciate ligament surgical procedures in Sweden. The registry includes both surgeon and patient reported data including patient, injury, and surgical characteristics as well as patient-reported outcome measures. While patient demographic characteristics, injury- and surgery-related factors are reported by the surgeon, questionnaires regarding current knee function are filled out by the patient. The registry has been described in more detail in previous literature [13, 14].

### Study population

Patients aged > 13 years undergoing primary ACLR with hamstring tendon autograft between 2005 and 2018 in the SNKLR were included. Patients with prior knee surgery, double-bundle ACLR, concomitant fracture (patella, femur, tibia, or fibula), other ligament injury, or neurovascular injury were excluded from further analysis. Fixation types “other”, “XO-button” as well as fixation types that are only used in ACLR with transtibial technique (Rigidfix, Transfix) were excluded because of the modern understanding of the superiority of the tibial-independent drilling technique at restoring the anatomy of the ACL [15–17]. Demographic data including patient age, sex, body mass index (BMI), and activity at time of injury were extracted from the SNKLR. Surgical information on concomitant injuries (meniscus, cartilage), time from injury to surgery, fixation types (Table 1) and subsequent revision ACLR were extracted. Activity performed at the time of injury was divided into six different groups: alpine/skiing, pivoting sport (American football/rugby, basketball, dancing, floorball, gymnastics, handball, ice hockey/bandy, martial arts, racket sports, soccer, volleyball, wrestling), non-pivoting sport (cross-country skiing, cycling, horseback riding, motocross/endure, skateboarding, snowboarding, and surfing/wakeboarding), other physical activity (other recreational sport, exercise, trampoline), traffic related, and other (other outdoor activity and work).

**Table 1:** Description of the fixation devices

**Femoral fixation**	
Metal interference screw	Direct tendon-to-bone interference fixation device
Endobutton	Continuous loop cortical suspensory fixation device (non-adjustable)
Staple	Titanium or stainless-steel compression tendon-cortical bone fixation device
AO screw	Graft sutures tied around stainless steel/titanium screw (i.e., Suture post)
Retrobutton	Continuous loop cortical suspensory fixation device (non-adjustable)
Retroscrew	Direct tendon-to-bone interference fixation device
Ezloc	Slotted femoral fixation device with cortical lever arm
Metal interference screw / Endopearl	Hybrid fixation device including direct tendon-to-bone interference fixation device and interlocking poly L-lactide ball
Toggleloc	Suspensory cortical fixation device (adjustable loop)
Tightrope	Suspensory cortical fixation device (adjustable loop)
Interference screw	Direct tendon-to-bone interference fixation device
Graftmax	Suspensory cortical fixation device (adjustable loop)
Ultrabutton	Suspensory cortical fixation device (adjustable loop)
**Tibial fixation**	
Metal interference screw	Direct tendon-to-bone interference fixation device
Intrafix	Direct tendon-to-bone interference fixation device using graft tensioner
Cobra	Graft sutures tied around stainless steel/titanium screw with washer
Staple	Titanium or stainless-steel compression tendon-cortical bone fixation device
Metal interference screw / Staple	Hybrid fixation device including direct tendon-to-bone interference fixation device with backup titanium or stainless-steel compression tendon-cortical bone fixation device
Endobutton	Continuous loop cortical suspensory fixation device (non-adjustable)
AO screw	Graft sutures tied around stainless steel/titanium screw (i.e., Suture post)
Retroscrew	Direct tendon-to-bone interference fixation device
Mitek anchor	Suspensory fixation with cortical anchor
Retrobutton	Continuous loop cortical suspensory fixation device (non-adjustable)
Resorbable screw	Direct tendon-to-bone interference fixation device
Metal interference screw / Osteosuture	Hybrid fixation device including direct tendon-to-bone interference fixation device with backup (transosseus) suture fixation
Tightrope	Suspensory cortical fixation device (adjustable loop)
Resorbable screw / Post	Hybrid fixation device including direct tendon-to-bone interference fixation device with graft sutures tied around stainless steel/titanium screw/staple for backup fixation
Suture washer	Graft sutures tied around stainless steel/titanium screw with smooth/spiked washer

The primary outcome was the ipsilateral revision rate by different femoral and tibial fixation device combinations, reported as “femoral fixation/tibial fixation” with backup fixation if applicable, within 2 years following primary ACLR. Revision rate within 2 years of the index procedure was chosen because ACLR fixation is most likely to contribute to ACLR revision in the first 2 years, during the process of graft maturation [18, 19]. Beyond 2 years, other factors such as tunnel position and patient activity level are likely more meaningful contributors to failure and thus revision.

### Statistical analyses

All statistical analyses were performed by using the SAS System for Windows (version 9, SAS Institute, Cary, North Carolina, USA). The count (n) and proportion (%) were used to present categorical variables, while continuous and ordinal data were presented by using the mean and standard deviation (SD), as well as the median with minimum and maximum.

## Results

### Baseline characteristics

Of the 23,238 patients included in this study, 13,087 (56%) were males (Table 2). The mean age at time of surgery was 27.3 years + 10.4, and the mean BMI was 24.5 + 3.3 kg/m^2^. The mean time from ACL injury to surgery was 19.1 + 33.7 months.

**Table 2:** Baseline characteristics of the study cohort

Variable	Total (*n* = 23,238)
Age at time of surgery (years), mean + SD, median (min-max)	27.3 + 10.4 25 (13–67)
BMI (kg/m^2^), mean + SD, median (min-max)	24.5 + 3.3 24.2 (15.4–49.8)
Sex (male), n (%)	13,087 (56.3)
Activity at time of injury, n (%)	
Alpine/skiing	3439 (14.8)
Pivoting sport	15,491 (66.7)
Non-pivoting sport	972 (4.2)
Other physical activity	890 (3.8)
Traffic related	357 (1.5)
Other	2083 (9.0)
Concomitant meniscus injury (yes), n (%)	
Lateral meniscus injury	5825 (25.1)
Medial meniscus injury	6201 (26.7)
Concomitant cartilage injury (yes), n (%)	5574 (24.0)
Lateral femoral condyle	1114 (4.7)
Medial femoral condyle	3889 (16.7)
Lateral patella	541 (2.3)
Medial patella	945 (4.1)
Lateral tibial plateau	1307 (5.6)
Medial tibial plateau	1009 (4.3)
Trochlea	647 (2.8)
Time from injury to surgery (months), mean + SD, median (min-max)	19.1 + 33.7 7.9 (0–551)
Revision, n (%)	584 (2.5)

*BMI *body mass index; *min-max* minimum-maximum; *SD* standard deviation; Pivoting sport (American football/rugby, basketball, dancing, floorball, gymnastics, handball, ice hockey/bandy, martial arts, racket sports, soccer, volleyball, wrestling); non-pivoting sport (Cross-country skiing, cycling, horseback riding, motocross/endure, skateboarding, snowboarding, and surfing/wakeboarding); other physical activity (**o**ther recreational sport, exercise, trampoline); other (other, outdoor activity and work)

The sums may vary due to missing data n (%): BMI 9725 (41.8), Activity at time of injury 6 (0.03); Details on the location of concomitant cartilage injury 17,695 (76.1); Time from injury to surgery 378 (1.6)

### Fixation types and revision rate

Endobutton and Tightrope were the most common femoral fixation devices (52.5 and 35.2%) (Table 3), while resorbable screw and AO screw were the most frequently used tibial fixation devices (24.3 and 22.9%). Of the included patients, 581 (2.5%) underwent revision surgery within 2 years after the primary ACLR.

**Table 3:** Fixation devices used in the study population

Variable	Total (*n* = 23,238)
Femoral fixation, n (%)	
Interference screw	3 (0.0)
Retroscrew	6 (0.0)
Staple	8 (0.0)
Metal interference screw / Endopearl	20 (0.1)
Graftmax	35 (0.2)
Ezloc	41 (0.2)
AO screw	59 (0.3)
Retrobutton	223 (1.0)
Toggleloc	307 (1.3)
Ultrabutton	313 (1.3)
Metal interference screw	1838 (7.9)
Tightrope	8181 (35.2)
Endobutton	12,204 (52.5)
Tibial fixation, n (%)	
Mitek anchor	1 (0.0)
Retrobutton	24 (0.1)
Cobra	36 (0.1)
Staple	375 (0.2)
Endobutton	92 (0.4)
Retroscrew	147 (0.6)
Suture washer	292 (1.3)
Metal interference screw / Osteosuture	519 (2.2)
Intrafix	1021 (2.2)
Resorbable screw / Post	646 (2.8)
Metal interference screw / Staple	1293 (5.6)
Tightrope	3900 (16.8)
Metal interference screw	4264 (18.3)
AO screw	5321 (22.9)
Resorbable screw	6645 (24.3)

#### Revision by combination of femoral/tibial fixation used in > 20 patients

**Fig. 1:**
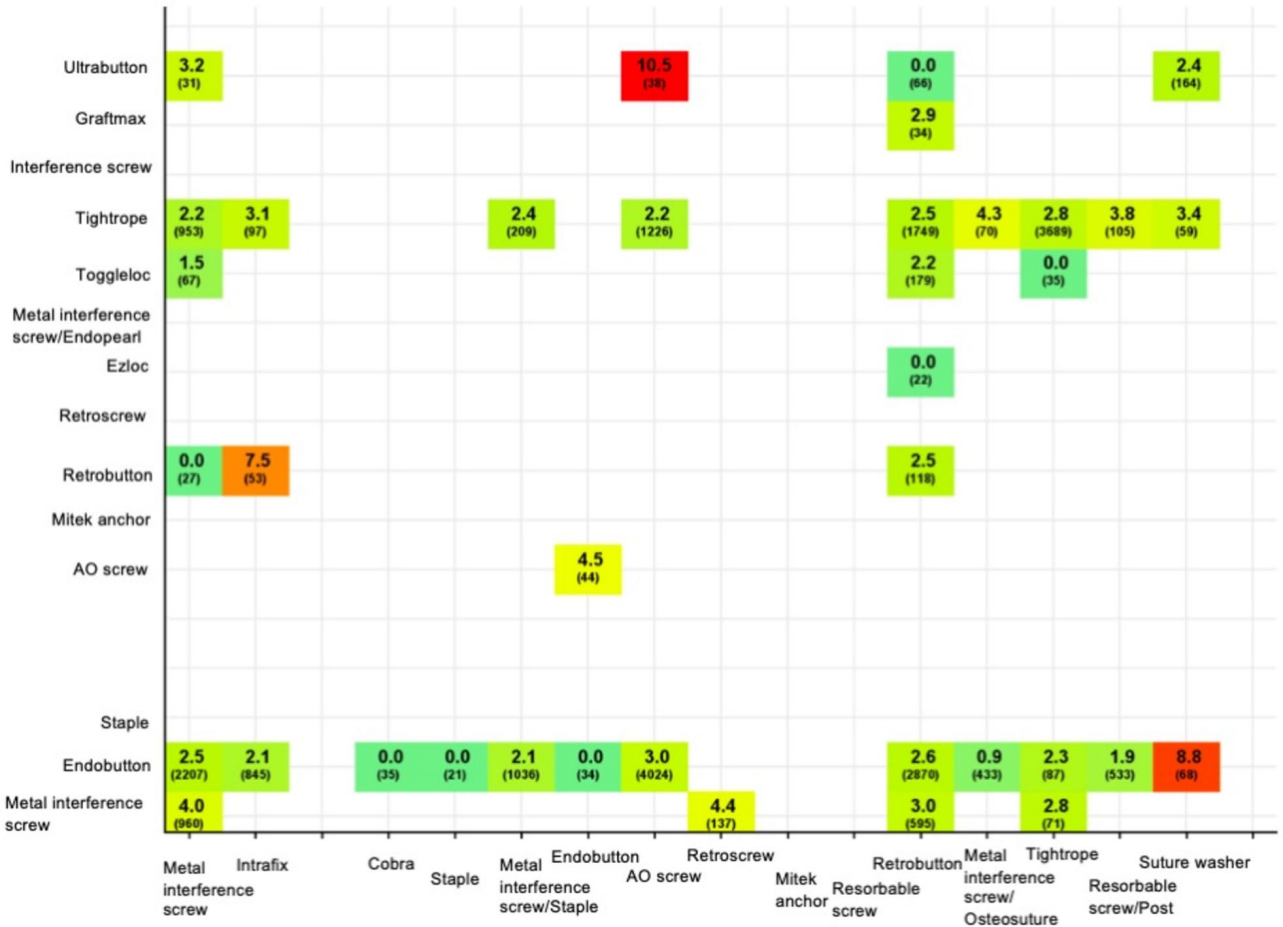
Revision within 2 years after ACLR, combinations of femoral/tibial fixation, *n* > 20 Revision rates by the specific femoral (y-axel) and tibial (x-axel) fixation combinations within 2 years after primary hamstring tendon autograft ACLR. The values are given as n and % for the total number and revision rate, respectively. ACLR = anterior cruciate ligament reconstruction

The most common combination of femoral/tibial fixation leading to revision within 2 years after primary ACLR was Ultrabutton/AO screw combination with a revision rate of 10.5% (Fig. 1). The Endobutton/Suture washer and Retrobutton/Intrafix combinations were also found to have high revision rates (8.8 and 7.5%, respectively).

#### Revision by combination of femoral/tibial fixation used in > 300 patients

**Fig. 2:**
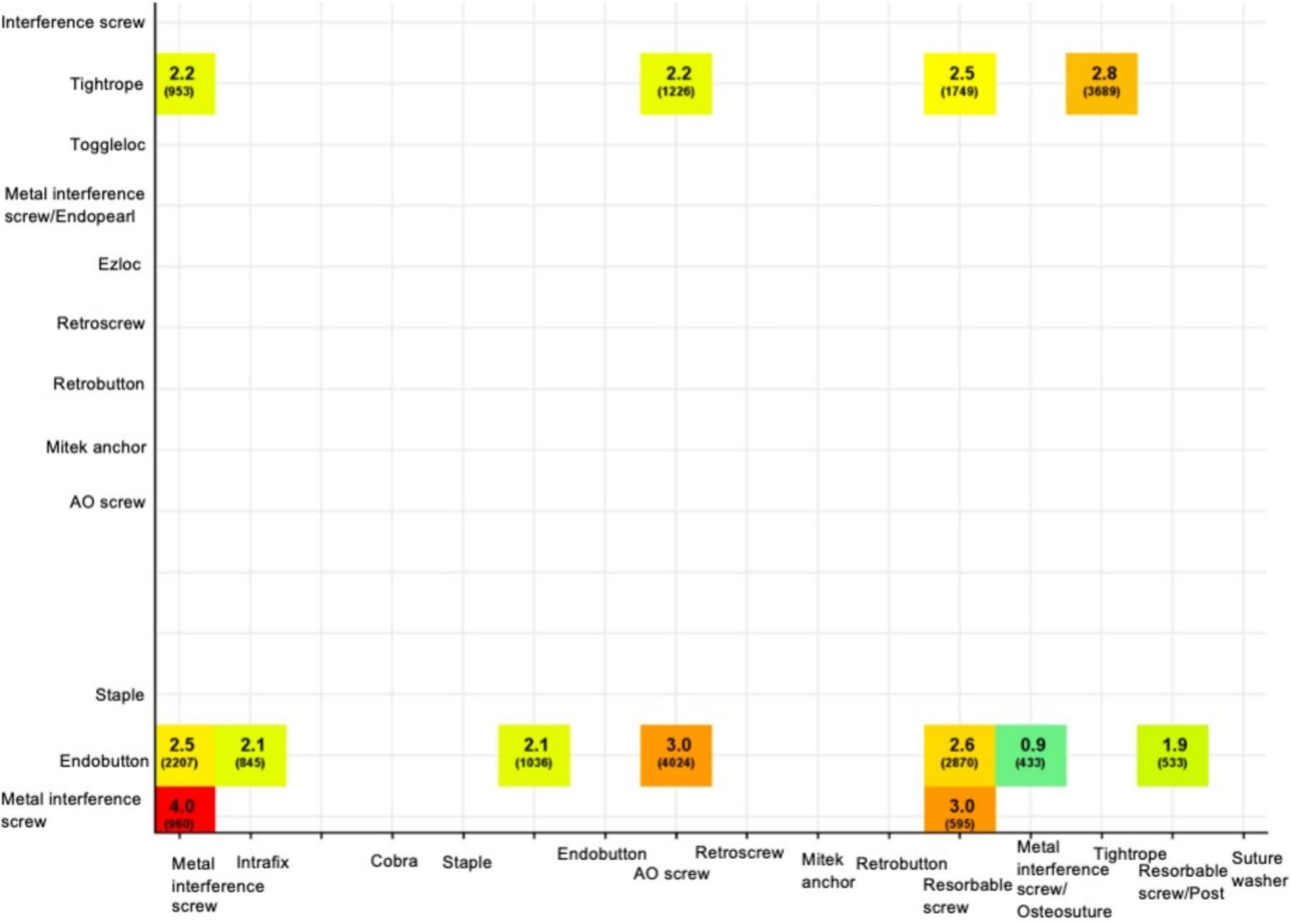
Revision within 2 years after ACLR, combinations of femoral/tibial fixation, *n* > 300 Revision rates by the specific femoral (y-axel) and tibial (x-axel) fixation combinations within 2 years after primary hamstring tendon autograft ACLR. The values are given as n and % for the total number and revision rate, respectively. ACLR = anterior cruciate ligament reconstruction

The most common fixation combination leading to revision was metal interference screw/metal interference screw followed by metal interference screw/resorbable screw and Endobutton/AO screw, with revision rates of 4.0, 3.0, and 3.0%, respectively (Fig. 2). The Endobutton/metal interference screw with backup Osteosuture fixation combinations, used in a total of 433 cases, had the lowest revision rate (0.9%). The Endobutton/resorbable screw with backup post fixation combination had the second-lowest revision rate (1.9%) and was used in a total of 533 cases.

## Discussion

The main finding of this study was that, among the most widely used fixation combinations, the greatest 2-year ACL revision rate was found when metal interference screws were used for both femoral and tibial fixation.

This study uniquely examines combinations of femoral- and tibial-sided fixation in a large volume of primary hamstring ACLR. While the majority of prior literature compares ACLR revision rates based on either femoral fixation or tibial fixation type in isolation, the evaluation of fixation combinations is an important contribution to our understanding of the relationship between fixation and ACLR failure. For example, aperture (i.e., interference screw) fixation is theorized to lead to a shorter mobile length of the graft, minimizing graft micromotion with potentially improved graft-tunnel healing [7, 8]. Graft fixation with both femoral and tibial interference screws would lead to the shortest mobile graft length, whereas fixation with one screw and one suspensory device would lead to a different mobile graft length. Therefore, the tibial-sided fixation could affect the biomechanical characteristics of the femoral-sided fixation. As another example, if interference screws risk damage to soft tissue grafts, [20, 21] then a construct with two interference screws would theoretically have a higher risk of graft revision than a construct with one screw. Once again, the combination of fixation devices matters more than fixation on one side alone.

Adding to previous literature, this study found that the metal interference screw/metal interference screw fixation combination resulted in a revision surgery most commonly among the more widely used fixation combinations. Previous literature shows varying effects of metal interference screw fixation on ACLR revision rates, for example with increased risk of revision ACLR with femoral metal interference screw fixation [9] and decreased revision risk with tibial metal interference screw fixation compared with other fixation types [22]. Furthermore, some previous literature has demonstrated lower stability rates and higher revision rates followed by ACLR with femoral aperture fixation compared to suspensory fixation [23, 24]. However, revision rates compared between the femoral and tibial metal interference screw combination versus other fixation combinations have not been reported, highlighting the original contribution of this study to the current literature.

The prominent revision rate seen with metal interference screws could be related to the metal threads cutting into the graft, thereby weakening it [20, 21]. This explanation is further supported by the minor revision rate in the current study with metal interference screw fixation combined with Osteosuture backup fixation, in which case the sutures may maintain graft fixation despite destruction of graft fibers by the cutting forces of the screw. In contrast, metal screws on both femoral and tibial sides, as opposed to one side only, may increase the risk of graft injury.

In contrast to previous studies, low revision rates were found in patients treated with femoral suspensory and tibial interference screw fixation combinations [11, 25, 26]. During the late 2000s and early 2010s, the use of anatomic ACLR surged in popularity, with a concurrent increase in the adoption of suspensory and interference screw fixation in tibial-independent anatomic ACLR [6, 27, 28]. Thus, the introduction of this new and complex technique may have involved a higher revision rate due to a learning curve and, subsequently, could have led to the increased revision rates with the fixation combinations frequently used in anatomic ACLR [29, 30]. However, improved outcomes have been observed over the recent years with anatomic ACLR, [29] which may partially explain our findings of low revision rates in patients with femoral suspensory and tibial interference screw fixations combinations. Examples of such fixation methods in the current study are the Endobutton/metal interference screw with backup Osteosuture fixation and the Endobutton/resorbable screw with backup post fixation combinations.

This study had strengths and limitations. Most importantly, the current study included a large patient sample and detailed information on 23,238 patients with ACLR. The SNKLR has previously been described to include data on > 90% of all ACLR performed in Sweden [6]. Consequently, the study population can be considered representative of the overall Swedish ACLR population. One limitation is that this study only included all soft tissue hamstring autograft, limiting the generalizability of the results to other graft choices. Second, this study defined failure as revision surgery, neglecting patients with poor functional outcomes who have clinically failed despite not undergoing revision surgery. The relatively long time from injury to surgery may affect the generalizability of the study results to populations undergoing earlier ACLR. Furthermore, this study did not include information on radiographic alignment, lateral extra articular procedures, postoperative rehabilitation protocols or complications, such as infection, as the data was not available from the registry. Finally, possible variation in concomitant injuries and their treatment were not assessed among the different fixation groups.

## Conclusion

Different early ACL revision rates were found across different combinations of femoral and tibial fixation devices within 2 years of primary hamstring tendon autograft ACLR. Metal interference screw fixation, particularly when performed on both the femoral and tibial sides, was found to result most commonly in a revision surgery. These findings may be helpful for surgeons in selecting appropriate fixation devices for hamstring ACLR.

### Supplementary Information

**Additional file 1.**

## Data Availability

The dataset analyzed during the current study are available from the corresponding author on reasonable request.
